# Extremophiles in an Antarctic Marine Ecosystem

**DOI:** 10.3390/microorganisms4010008

**Published:** 2016-01-11

**Authors:** Iain Dickinson, William Goodall-Copestake, Michael A.S. Thorne, Thomas Schlitt, Maria L. Ávila-Jiménez, David A. Pearce

**Affiliations:** 1 Department of Applied Sciences, Faculty of Life Sciences, Northumbria University, Ellison Building, Newcastle-upon-Tyne NE1 8ST, UK; iain.dickinson@northumbria.ac.uk; 2 British Antarctic Survey, Natural Environment Research Council, High Cross, Madingley Road, Cambridge CB3 OET, UK; wgco@bas.ac.uk (W.G.-C.); mior@bas.ac.uk (M.A.S.T.); thomas.schlitt@novartis.com (T.S.); 3 Prudhoe St., Alnwick NE66 1UG, UK; mlavilaj@gmail.com; 4 The University Centre in Svalbard (UNIS), P.O. Box 156, Svalbard, Longyearbyen N-9171, Norway

**Keywords:** Antarctica, bacteria, biodiversity, metagenome, polar, marine, bioprospecting, fosmid, extremophile, rare

## Abstract

Recent attempts to explore marine microbial diversity and the global marine microbiome have indicated a large proportion of previously unknown diversity. However, sequencing alone does not tell the whole story, as it relies heavily upon information that is already contained within sequence databases. In addition, microorganisms have been shown to present small-to-large scale biogeographical patterns worldwide, potentially making regional combinations of selection pressures unique. Here, we focus on the extremophile community in the boundary region located between the Polar Front and the Southern Antarctic Circumpolar Current in the Southern Ocean, to explore the potential of metagenomic approaches as a tool for bioprospecting in the search for novel functional activity based on targeted sampling efforts. We assessed the microbial composition and diversity from a region north of the current limit for winter sea ice, north of the Southern Antarctic Circumpolar Front (SACCF) but south of the Polar Front. Although, most of the more frequently encountered sequences  were derived from common marine microorganisms, within these dominant groups, we found a proportion of genes related to secondary metabolism of potential interest in bioprospecting. Extremophiles were rare by comparison but belonged to a range of genera. Hence, they represented interesting targets from which to identify rare or novel functions. Ultimately, future shifts in environmental conditions favoring more cosmopolitan groups could have an unpredictable effect on microbial diversity and function in the Southern Ocean, perhaps excluding the rarer extremophiles.

## 1. Introduction

Marine microbial communities have been shown to present small-to-large scale biogeographical patterns worldwide [1], debunking the previous view of a rather homogeneous microbial landscape lacking the gradients seen for macroorganisms [2]. In fact, free-living marine microbial community composition can differ significantly between locations and these differences may correlate with environmental factors [3,4] such as salinity [5,6], depth [7,8,9,10] and the presence of ocean fronts [11].

Recent attempts to explore marine microbial diversity and the marine microbiome have shown a large proportion of previously unknown diversity worldwide [12,13]. In particular, the Southern Ocean is known to comprise the largest proportion of unknown genes to date [13]. From such studies 95%–35% of the bacterial biomass described from diatom assemblages living under the ice are estimated to be psychrophilic [14]. However, most of the reports on true psychrophiles from the marine ecosystem are restricted to sea-ice assemblages [15,16] or relatively deep sediment [17,18], while studies on Subantarctic and Antarctic heterotrophic bacterial isolates have shown substantial bacterial growth rates over a wide range of temperatures (0 to 20 °C [19,20,21,22,23], *i.e.*, wider than those found to occur at this location). This uneven distribution of psychrophilic organisms (or more specifically their reports in the literature) highlights the likeliness of large variations in the structure and composition of marine bacterioplankton communities at relatively small spatial and temporal scales, as previously suggested [24,25].

Large scale sequencing studies can identify the number of functions present in a sample, but not the type of function unless similar sequences have already been characterized and deposited in curated databases. On the other hand, blind functional exploration such as that based on metagenomic techniques, where sections of unknown sequences are cloned into fosmids and subsequently characterized, allows the identification and expression of function regardless of the sequence responsible. Thus, in an era of increasing effort aimed at fully sequencing all genes present in the environment, direct expression based studies remain a highly efficient technique for large-scale functional bioprospecting.

The Antarctic marine environment has been traditionally mined for potentially useful enzymes and antimicrobial activity using culture based systems [26,27,28,29]. However, despite the potential of metagenomic technologies as bioprospecting tools, these techniques have had limited application in Antarctic marine ecosystems to date, and mainly look at differences in population diversity [30,31,32], primary production [33], cold adaptation [34] and secondary metabolism [35].

**Figure 1:**
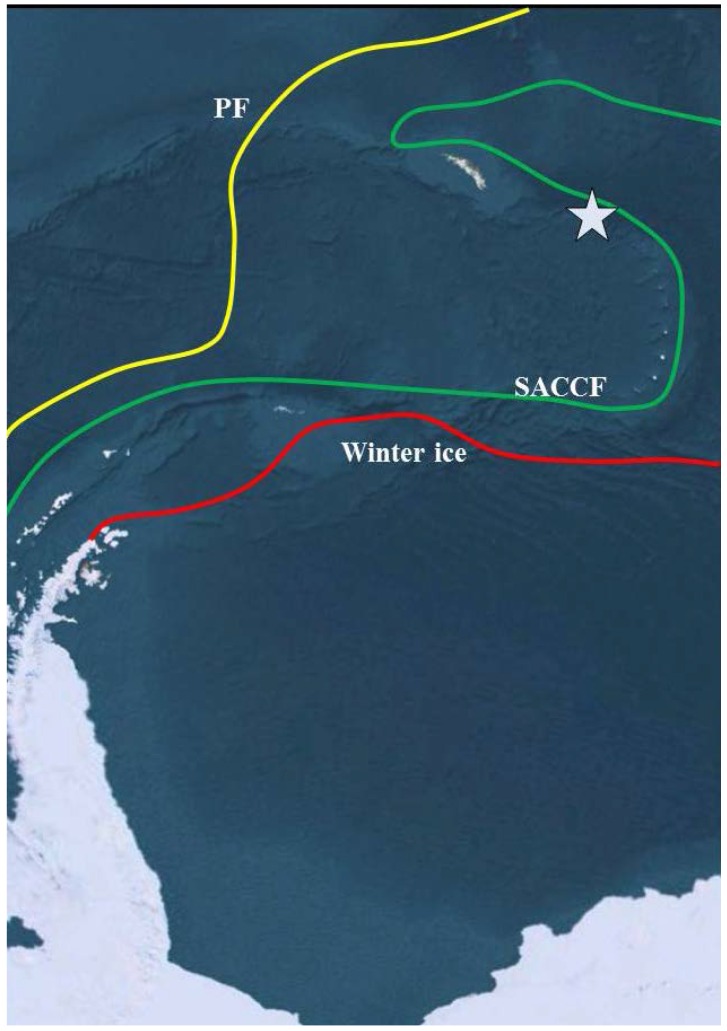
Sampling location. The star shows the approximate location of the sampling point in relation to the Southern Antarctic Circumpolar Front (SACCF, in green), the Polar Front (PF, in yellow) and the maximum winter sea ice recorded in 2011 (Winter ice, in red). Antarctic winter sea ice data for 2011 from the Weddell Sea were obtained from climate.gov. The approximate locations of the Polar Front and the Southern Antarctic Circumpolar Front are a reproduction from Talley *et al.*, (eds.) Descriptive physical oceanography: An Introduction. Elsevier, 2011 [37]. The background map is derived from Google Earth.

Here, we explore the potential of metagenomic analysis as a targeted bioprospecting tool. To this end, we selected an Antarctic boundary region and transition zone in the vicinity of South Georgia (Figure 1), north of the Southern Antarctic Circumpolar Front (SACCF), south of the Polar Front and north of the islands in an area known to experience a large annual phytoplankton bloom as a result of nutrient upwelling [36]. Thus, this study not only presents a new perspective on biodiversity in the Southern Ocean, but also identifies a large number of potential targets for bioprospecting from a single sampling effort.

## 2. Experimental Section

### 2.1. Site Description and Sampling

Surface water samples were collected during the Austral summer of 2006 between South Georgia and the South Scotia Arc (53.3° S, 46.9° W; Figure 1) [37]. This region is south of the Polar front and north of both the northern current boundary of winter sea ice and the Antarctic Circumpolar Current. In addition, there is a large annual phytoplankton bloom to the north of the islands as a result of nutrient upwelling [36]. We collected 300 L of seawater 30 m below the surface at the chlorophyll maximum using a CTD. The sample water was passed through a sonication bath to disrupt suspended particles and through a 2.7 µm prefilter using a sterile stainless steel housing. The water was then subjected to ultrafiltration in a Pellicon II ultrafiltration system with 1 × 10,000 Da molecular weight cut-off filters. The permeate was excluded and the retentate recycled through the system until the total seawater volume reached <300 mL, and frozen at −20 °C for further analysis.

### 2.2. Metagenomic Library Construction

The 300 mL retentate was defrosted slowly and centrifuged at 38,000 revolutions per minute (r.p.m.) to recover whole cells in a centrifugal concentrator. The cell suspension was used to make agarose plugs using plug molds and high molecular weight cut off agarose at 55 °C. Community genomic DNA was extracted directly from whole cells embedded in agarose by digestion with lysozyme in TAE for 35 min at 37 °C. Pellets were suspended in 0.1 mL TE buffer and incubated at 4 °C for 16 h. Subsequently, 0.1 mL of loading buffer was added and the samples were run on a 1% low melting point agarose gel at 20 V overnight, along with a size standard of ~40 kbp. Bands of the appropriate size were removed with a sterile scalpel. Enzymes were inactivated by heating the mixture to 60 °C for 30 s. DNA was recovered from the gel using Gelase. The DNA was then precipitated, pelleted and resuspended in 4.5 µL of TE buffer, and the concentration was determined using known standards on a 1.5% agarose at 120 V for 1 h. Aliquots of 12 µL were made up with a concentration of 0.5 µg·µL^−1^. DNA was sheared using a hypodermic syringe and end repaired by adding 1.85 µL of 10× end repair buffer, 1.85 µL of 2.5 mM dNTP mix, 1.85 µL of 10 mM ATP and 0.92 µL of end repair enzyme mix to the 12 µL of DNA. This mixture was incubated at room temperature for 40 min, followed by 10 min at 70 °C. The DNA was then ligated into pEpiFOS-5 vectors by adding 2 µL of sterile water, 3 µL of 10× Fastlink ligation buffer, 3 µL of 10 mM ATP solution, 3 µL Fastlink ligase and 1 µL of the pEpiFOS-5 vector. The mixture was incubated at room temperature for 2 h, followed by 10 min at 70 °C. EPI100-T1 plating strain of *E. coli* was transformed to contain the fosmids using Lambda phages as per the manufacturers’ instructions (Epicentre, Madison, WI, USA). The cells containing the fosmids were selected by streaking the plating strain solution onto Luria broth (LB) plates containing chloramphenicol. Single colonies were picked into individual wells of a 96 well plate, containing 40 µL of LB and 12.5 µg·mL^−1^ chloramphenicol. Plates were stored at −80 °C until analyzed. Quality control was established by end sequencing 20 random fosmids using pEpiFOS-5 forward and reverse end sequencing primers (Epicentre, Madison, WI, USA) to ensure environmental DNA had been successfully incorporated, and that this had come from microorganisms that one might expect to find in this extreme environment. In microbiology, results can sometimes be constrained by the analysis technique, so this study used two independent high throughput sequencing methodologies, 454 pyrosequencing and MiSeq.

### 2.3. 454 Pyrosequencing

Cells from 25 plates (10% of the total) were combined (to favor depth of sequencing rather than coverage) and cultured in LB with 12.5 μg·mL^−1^ chloramphenicol overnight in a shaking incubator at 37 °C until an OD of 0.8 was obtained. The cells were centrifuge-concentrated and used to construct a metagenomic library for 454 pyrosequencing. Fosmids were extracted from *E. coli* cells using the QIAGEN Plasmid Midi Kit (QIAGEN Plasmid Midi Kit, Cat. No. 12145. QIAGEN, Manchester, UK) and then treated with ATP-dependent exo-nuclease (Plasmid-Safe^TM^ ATP-Dependent DNase, 10 U·µL^−1^ 10,000 U, Cat. No. E3110K, Epicentre, Madison, WI, USA). An amplicon library was generated using the Rapid Library Preparation kit and following the manufacturer’s recommendations in the GS FLX Titanium Series Rapid Library Preparation Method Manual (Roche, Branford. CT, USA). Briefly, the PCR amplicons were purified using AMPure beads (Agencourt Bioscience Corporation, Beverly, MA, USA), adaptors were blunt-end ligated to the fragment and the dsDNA amplicon library was quantified via fluorometry using Quanti-iT Pico Green reagents (Invitrogen, Carlsbad, CA, USA). The library was then subjected to clonal amplification by emulsion PCR followed by pyrosequencing on a 454 GS FLX sequencer according to the manufacturer’s instructions (NEB NextQuick 454 library prep kit E6090, Hitchin, Herts, UK).

### 2.4. MiSeq Sequencing

Fosmids were extracted using FosmidMAX DNA purification kit (Epicentre, Madison, WI, USA) and sequenced using an Illumina MISEQ sequencing system at the Centre for Genomic Research, Liverpool.

### 2.5. Data Analysis

The 454 pyrosequencing sequencing reads were trimmed to remove library preparation related and low quality sequence reads using both Geneious [38,39] and Mothur [40] before systematic artifacts resulting from the 454 pyrosequencing technology were removed [41]. Quality trimmed sequences greater than 100 bp in length were submitted to the MG-RAST [42] metagenomic server where they were analyzed against the SILVA [43], Greengenes [44] and RDP [45] 16S ribosomal DNA databases using an e-value cut-off of 1e-10 with minimum identity cut-off of 60% and a minimum alignment length cut-off of 15. Alpha diversity, based on an information theoretic metric, was calculated from the homology assignments of the 16S RNA gene sequences, as outlined in the MG-RAST pipeline. The MiSeq generated reads were trimmed for the presence of Illumina adapter sequences using Cutadapt version 1.2.1 (MIT, Cambridge, MA, USA), and were further trimmed using Sickle version 1.200 with a minimum window quality score of 20. Reads shorter than 10 base pairs after trimming were removed. Trimmed data were then assembled with CLC Genomic Workbench 5.1.1 and were annotated into an open source server MG-RAST [42] to study the taxonomic composition and abundance of bacterial species in the samples. The maximum e-value for a significant match was set to 1e-5, the minimum alignment length was set to 50 bp and the minimum identity cut-off was set at 97% as recommended by Meyer *et al.* [42] and Andersson *et al.* [46].

### 2.6. Screening of Libraries with PCR Amplification to Identify Specific Functions

Primers were used to identify specific sequences from the fosmid library. The metagenomic library was screened using a range of primers for viral (Cyanophage CPS4GC, CPS5 [47,48] and Phycodnaviridae AVS1, AVS2 [49]), fungal (ITS1F/ITS4F; [50,51]), phosphonate [52] and nitrogen cycling (nosZ-F/nosZ-R, nirS1F/nirS6R and nifHF/nifHRb; [53,54]) genes. A selection of *E. coli* cells containing fosmids were screened for antibiotic production.

## 3. Results and Discussion

### 3.1. Quality Control

End sequencing of two partial reads at both ends of the insert of 20 fosmids were used for quality control. The size of the insert DNA was 35,000–45,000 bp. These sequences matched known clones belonging to three main categories, all suggestive of a marine origin: those similar to Global-Ocean-Sampling (GOS) clones, marine metagenome genomic clones and those similar to an Ionian Sea marine metagenome (Supplementary Table S1)). In addition, we found matches with sequences common to the marine bacteria (Candidatus *Pelagibacter ubique* HTCC1062 and *Roseobacter denitrificans* OCh 114) and commensal with marine organisms (*Strongylocentrotus purpuratus* clone, Purple Sea Urchin). This suggested that the metagenome creation was successful and the fosmids matched data in various marine metagenomes including sequences from typical marine bacteria and some eukaryotes.

### 3.2. Preliminary Analysis

In total, 65,536 of the 1.5 million reads obtained through 454 pyrospequencing that contained a partial 16S rRNA sequence were analysed. Of these, 18,155 classifications were identified as 16,570 singletons, 482 doubletons, 331 identifications made more than 10 times and 29 identifications made more than 100 times. Of these classifications, 30% represented uncultivated bacteria, 30% represented bacteria that could be identified to genus level and 30% represented bacteria that could be identified to species level (including 300 type strains). Matches ranged from 10 bp at 95% to 400 bp at 100%. The relationship between sampling effort and new OTU discovery was still linear at the OTU level, but approached asymptote when identified to only the genus level. The total number of sequences containing taxonomical information from the MiSeq data was 625,519. Blasting these sequences in the SEED database produced 2379 phyla, the top 25 by abundance for each is shown in Table 1.

**Table 1:** Top 25 species in 454 and MiSeq data by abundance.

454 Species	MiSeq Species
*Ruegeria pomeroyi* (27,024)	*Pseudomonas aeruginosa* (21,810)
*Roseobacter denitrificans* (23,469)	Candidatus *Pelagibacter ubique* (17,625)
Candidatus *Pelagibacter ubique* (22,434)	*Roseobacter denitrificans* (16,349)
*Rhodobacter sphaeroides* (15,361)	Rhodobacterales bacterium HTCC2255 (15,625)
*Ruegeria* sp. TM1040 (14,489)	*Ruegeria pomeroyi* (13,319)
*Saccharophagus degradans* (14,300)	*Saccharophagus degradans* (11,712)
*Pseudomonas aeruginosa* (13,720)	*Ruegeria* sp. TM1040 (11,029)
*Dinoroseobacter shibae* (13,607)	*Octadecabacter antarcticus* (8624)
*Congregibacter litoralis* (11,834)	*Rhodobacter sphaeroides* (8519)
*Pseudomonas fluorescens* (10,692)	*Cellvibrio japonicus* (8484)
*Roseovarius* sp. 217 (9962)	*Congregibacter litoralis* (8400)
*Roseobacter* sp. MED193 (9629)	*Dinoroseobacter shibae* (7417)
*Pseudomonas putida* (9261)	*Jannaschia* sp. CCS1 (7057)
*Roseovarius nubinhibens* (8753)	*Teredinibacter turnerae* (6085)
*Loktanella vestfoldensis* (8319)	*Roseovarius nubinhibens* (5549)
*Cellvibrio japonicus* (8229)	*Roseovarius* sp. 217 (5291)
*Hahella chejuensis* (6977)	*Roseobacter* sp. MED193 (5136)
*Sulfitobacter* sp. NAS-14.1 (6741)	*Loktanella vestfoldensis* (4961)
*Colwellia psychrerythraea* (6581)	Marine gamma proteobacterium HTCC2143 (4509)
*Oceanicola batsensis* (6479)	*Roseobacter* sp. GAI101 (4494)
*Marinobacter hydrocarbonoclasticus* (6247)	Rhodobacteraceae bacterium HTCC2083 (4292)
*Sulfitobacter* sp. EE-36 (6111)	*Marinobacter hydrocarbonoclasticus* (4258)
*Pseudomonas mendocina* (6090)	*Hahella chejuensis* (4245)
*Maritimibacter alkaliphilus* (6010)	Candidatus *Puniceispirillum marinum* (4187)
*Pseudoalteromonas atlantica* (5972)	Marine gamma proteobacterium HTCC2148 (4100)

### 3.3. Taxonomy from 454 Pyrosequencing Data

**Figure 2:**
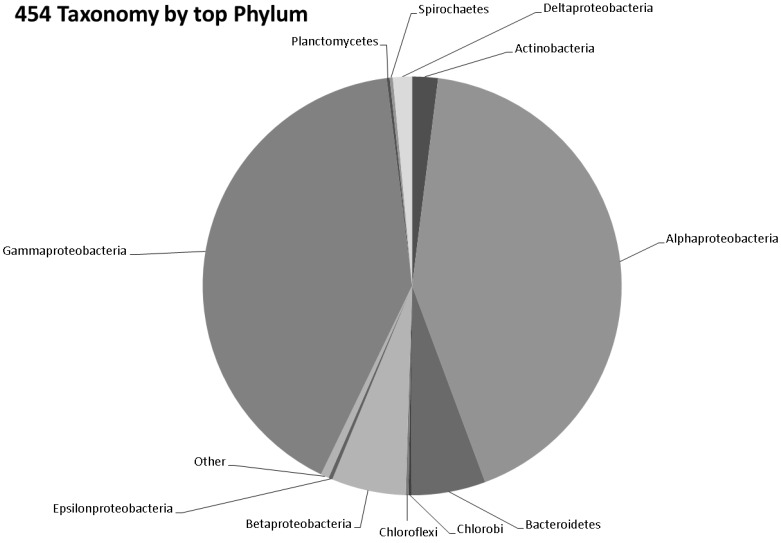
Pie chart representing the number of bacterial genera identified by 454 pyrosequencing.

**Figure 3:**
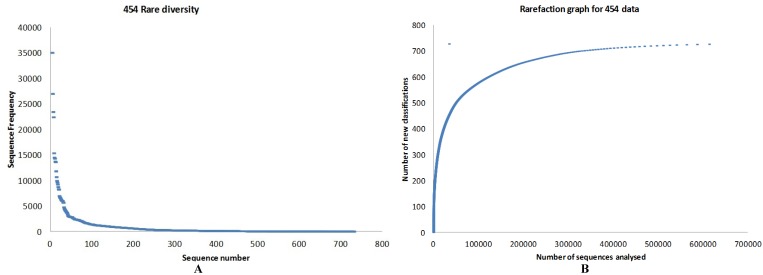
(**a**) Rare diversity and (**b**) rarefaction estimated based on 454 data analysed.

The number of bacterial genera (314) identified by 454 pyrosequencing are given in Figure 2 and Table S2. Of the 600 bacterial species identified in the 454 data, the top 25 species by abundance are given in Table 1. Screening the 454 data for archaeal sequences gave 35 Euryarchaeota, 13 Crenarchaeota and one each of Nanoarchaeota, Thaumarchaeota and Korarchaeota (Table S3). Screening 454 pyrosequencing based metagenome data for viral sequences gave 42 unclassified Caudovirales phage like viruses and two unclassified viruses. This included 19 Podoviridae, 10 Myoviridae, 10 Siphoviridae, one Mimiviridae and one Phycodnaviridae. A rarefaction analysis of the data is given in Figure 3a,b. Rare diversity was calculated using frequency distribution that showed that the most common individual sequence match in 454 was *Ruegeria pomeroyi*, which occurred 27,024 times, and that only 12 species were identified once.

### 3.4. Gene Ontology from 454 Pyrosequencing Data

**Figure 4:**
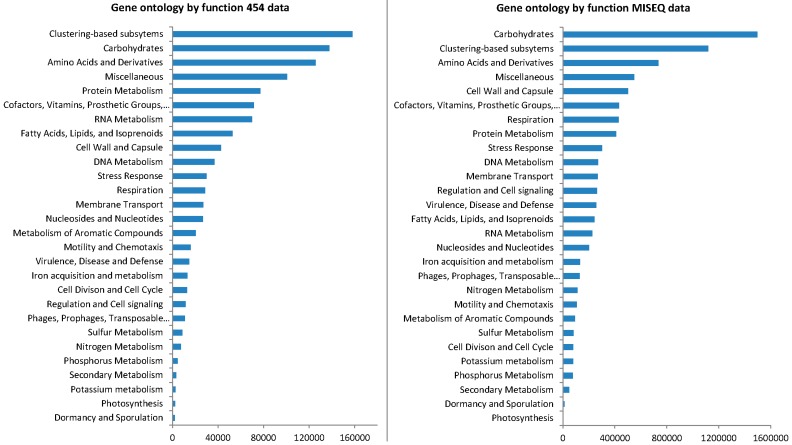
Gene Ontology by function.

After removing the housekeeping genes and those used in primary metabolism (carbohydrate, protein genes, *etc.*), which accounted for 90% of genes identified (Figure 4; Table S4), the following functions had the highest abundance in the 454 data (with percentage of abundance shown in parentheses); flagellar motility in prokaryota (1.37%), oxidative stress (1.15%), resistance to antibiotics and toxic compounds (0.83%), phages and prophages (0.66%), iron acquisition (0.6%), osmotic stress (0.57%), ammonia assimilation (0.54%), heat shock (0.52%), phosphate metabolism (0.29%) and organic sulfur assimilation (0.28%). A relatively small number of genes could be directly attributed to secondary metabolism and/or the ecological function within the environment: virulence, defense and disease, iron acquisition and metabolism, cell division and cell cycle, regulation and cell signaling and phages, prophages transposable elements and plasmids; in particular, genes pertaining to sulfur metabolism, nitrogen metabolism, phosphorous metabolism, potassium metabolism, dormancy and sporulation, secondary metabolism, and photosynthesis.

### 3.5. Taxonomy from MiSeq Data

**Figure 5:**
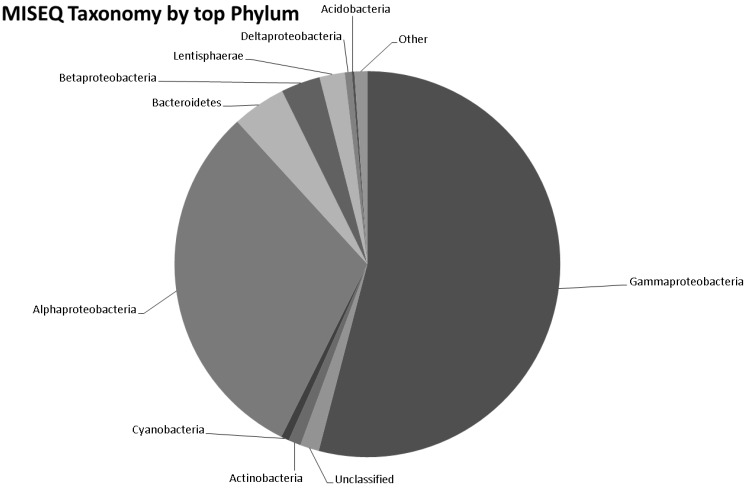
Pie chart representing the number of bacterial genera identified by MISEQ.

**Figure 6:**
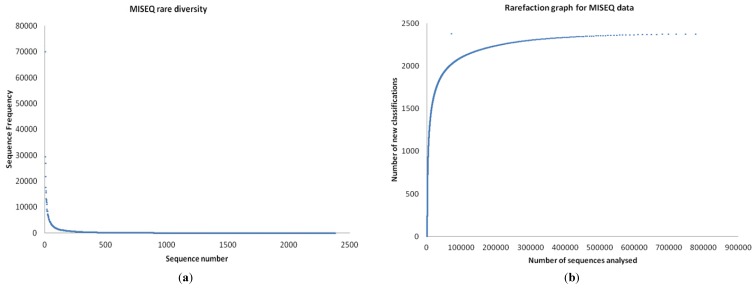
**(a)** Rare diversity and **(b)** rarefaction estimate based on MISEQ data analysed.

The number of bacterial genera by MISEQ (526) is given in Figure 5 and Table S5. Of the 1824 species of bacteria identified in the MISEQ data the top 25 species by abundance are given in Table 1. Screening the MISEQ data for archaeal sequences showed 54 Euryarchaota, 17 Crenarchaeota, five Thaumarchaeota, one Korarchaeota and six unclassified archaeal sequences (Table S6). Screening the MISEQ data for viral sequences gave 107 unclassified Caudovirales, including; 44 Siphoviridae, 37 Myoviridae, 25 Podoviridae and one unclassified. It also gave 42 uncultured phage-like virus sequences and 13 unclassified viral sequences. A rarefaction analysis of the data is given in Figure 6a,b. Rare diversity was calculated using frequency distribution that showed that the most common individual sequence match was *Pseudomonas aeruginosa*, which occurred 21,810 times, and that 219 species were identified once.

### 3.6. Gene Ontology from MiSeq Data

After removing the housekeeping genes and those used in primary metabolism (Figure 4; Table S4), which equaled 85% of genes in the MISEQ data, the following functions had the highest abundance; resistance to antibiotics and toxic compounds (2.28%), oxidative stress (1.54%), phages and prophages (1.28%), flagellar motility in prokaryota (0.98%), siderophores (0.7%), osmotic stress (0.53%), organic sulfur assimilation (0.43%), inorganic sulfur assimilation (0.33%), heat shock (0.26%) and aromatic amino acids and derivatives (0.24%). A relatively small number of genes could be attributed to secondary metabolism and/or the ecological function within the environment: iron acquisition along with phages, prophages, transposable elements and plasmids, nitrogen metabolism, motility and chemotaxis and metabolism of aromatic compounds. In particular, genes classified as dormancy and sporulation, sulfur metabolism, potassium metabolism, phosphorus metabolism, secondary metabolism and photosynthesis.

It is becoming commonly accepted that biodiversity of bacterioplankton is composed of abundant taxa that perform most of the ecosystem function and rarer, presumably less active taxa [5]. Data collected in this study support this, as the top 25 species represents 42% of the total sequence abundance in the 454 data and top 25 species represents 35% of the total sequence abundance in the MISEQ data.

### 3.7. Patterns of Bacterial and Functional Diversity

Microbial communities, and in particular those in Polar ecosystems tend to be dominated by a relatively restricted group of more numerous taxa [55,56]. In addition to those, a large number of taxa of low abundance constitute “rare diversity”. Here we aimed to target an ecosystem expected to be rich in diverse extremophiles. However, most strains found within our data were not obligate psychrophiles and belonged to a relatively limited number of genera. Indeed, the list seemed to be typical marine bacteria and bacteria able to cope with relatively low temperatures and marine salinity (*Pseudomonas* is a good example). In fact, the dominant groups are mainly cosmopolitan generalist groups, such as *Rosebacter* common from marine systems elsewhere in the world. While extremophiles were rare, they were also diverse.

From our data, we could identify a high proportion (85-90%) of functional housekeeping genes related to cell growth and metabolism; however, a proportion of the functional genes found might be expected to have a direct effect on the immediate environment. This dominance of housekeeping genes would suggest a disproportionate importance of the few key genes that determine which species come to dominance. However, we still know relatively little about what controls the distribution and dominance of keystone microbial species, but the data presented here suggests that finding those key genes could be an achieveable goal.

Most of the dominant groups identified also shared involvement in the nitrogen cycle as a common feature, suggesting that nitrogen might be a limiting factor in this environment. This follows previous studies which show that that nitrogen could be limiting in the Southern Ocean [57]. Our analysis potentially supports this theory as *Roseobacter denitrificans* was particularly significant here. In the Antarctic coastal environment, Lo Gludice *et al.* [58] were able to show that the genera *Psychromonas* and *Pseudoalteromonas* accounted for 65.4% of total isolates and were ubiquitous, thus suggesting that they may play a key role within the analyzed bacterioplankton community. They point out that, *Pseudoalteromonas* isolates, in particular, possess nitrate reductase and are able to hydrolyze substrates for protease, esterase, and β-galactosidase, thus indicating their involvement in the carbon and nitrogen cycles. Bacteria also play an important role in pelagic food webs [59]; for example, by redispersing silica [60], can produce iron-binding ligands [61] and can significantly affect the proliferation of phytoplankton. A high level of competition among bacterial strains might be inferred through the proportion of dominant groups who are involved in the production of secondary metabolites, also key for bioprospecting. Interestingly, we could identify quorum sensing and antibiotic production as relevant examples of secondary metabolism in our dataset.

Our data showed a large proportion of unexplored diversity and functional potential, as ~10% of the sequences did not match known functions in sequence databases. Thus, the metagenomic library built here harbors enough novel variability of function to maintain the exploration for functions of industrial and technological application without the need of additional sampling effort. Moreover, the large diversity of rare species found, in particular among extremophiles, identified this group as an interesting target for bioprospecting.

### 3.8. Viral Community

Compared to bacterial communities, viral abundance was surprisingly low in our study, although the Myoviridae and the Podoviridae were also found by Grzymski *et al.* [30]. It is noteworthy that the concentration of viral DNA in the sample was surprisingly low (0.6%). Given the numerical superiority of viruses in microbial communities and the importance of bacteriophage DNA in bacterial evolution, this suggests that a component of the approach and methodology selectively excludes viral DNA, the cloning procedure selects for prokaryote DNA, the bioinformatic pipeline removes viral DNA or we have yet to fully understand the structure and function of viral DNA in bacterial chromosomes from such extreme environments.

### 3.9. Cyanobacteria

Cyanobacteria are phototrophs and therefore important in the carbon cycle; they are generally absent from the Southern Ocean and methodologically often underrepresented in this type of analysis. Only 0.83% of the sequences described belonged to cyanobacteria (Table S7). Although this is different from other parts of the ocean where cyanobacteria are abundant, cyanobacteria are not common in Polar marine waters.

### 3.10. Archaea

Very few Archaea were found in the sequence analysis (809 sequence hits representing 83 different species). This could be symptomatic of the profile of DNA sequences available in the sequence databases, due in part, to the relative difficulty of culturing and sequencing archaeal genomes from the Southern Ocean. However, more likely they are not abundant in this environment, although, Delong *et al.* [62] showed that Antarctic seawater samples contained exceptionally high amounts of archaeal rRNA (about 18%–30% of the total picoplankton rRNA). Sea-ice bacteria [63] as well as archaeal assemblages [64] were shown to include diverging SSU rDNA variants from geographically separated areas, although some identical strains were also found. Archaeal communities were found to be not as diverse as the bacterial communities in an Antarctic polynya, and marine group I was dominant (>80%) [65] and virtually absent in summer surface waters, but were present in summer deep and winter surface samples [66]. The Archaea that were there could be divided into two groups: the halophiles and the methanogens. Extremophiles were evident within this group. Halophilic archaea included *Haloarcula marismortui*, *Halogeometricum borinquense*, *Natronomonas pharaonis* and *Haloferax volcanii*. Methanogens included *Methanococcus maripaludis*, *Methanothermobacter*, *Methanosarcina mazei* (which has a diverse metabolism), *Methanosarcina acetivorans* (found in diverse environments), *Methanosarcina barkeri*, *Methanobrevibacter smithii* and *Haloquadratum walsbyi*. Interestingly, they cover the range of temperature optima, from *Methanococcoides burtonii*, an extremophile that prefers the extreme cold, through *Methanococcus maripaludis* which is mesophillic, with an optimal growth temperature of 35–40 °C and the thermophile *Methanothermobacter thermautotrophicus* to *Pyrococcus horikoshii*, which thrives at an optimal temperature of 98 °C and the extreme hyperthermophile *Ignicoccus hospitalis*.

### 3.11. Actinobacteria

In the Antarctic terrestrial ecosystem, Actinobacteria are dominant. We are interested in the presence of Actinobacteria, as they are traditionally associated with soil samples and soils are traditionally targeted for secondary metabolite production. In the Antarctic coastal environment in Terra Nova Bay, Lo Giudice *et al.* [58] suggested that the ability of the Actinobacteria to survive and proliferate in the seawater was because they generally showed a wide range of salt tolerance and appeared to be particularly competitive with strictly marine bacteria by better utilizing supplied carbon sources. However, this appears not to be the case for the marine ecosystem, despite terrestrial run-off, as they were relatively low frequency (1.9%). However, this could mean that the functional significance of the ones observed could be disproportionate.

### 3.12. Methodological Potential in Bioprospecting

The MISEQ generated four times more sequences than the 454 pyrosequencing, so one would expect the greater number of sequences to show a better coverage of the population. Indeed, the MISEQ sequencing revealed a much greater diversity of taxa than the 454. This included 212 more genera covering around 1224 more species than the 454 data analysis. It even revealed phyla that the 454 sequencing did not detect, including; Chysiogenetes, Nitrospirae and Zeta proteobacteria, which were all detected, albeit in low numbers, by the MISEQ sequencing. The top 25 species in both sequencing types were typically uniform, with the most represented species occurring on both lists, although in a slightly different order. The 454 pyrosequencing analysis only detected 12 bacterial species occurring only once, whereas the MISEQ data showed 219 occurrences of species only being detected once. Therefore it might be that the MISEQ sequencing protocol is better at detecting the rare taxa in an environment than the 454, whilst maintaining a good representation of the abundant dominant taxa as the 454 pyrosequencing.

### 3.13. Ecological Characteristics of the Microbial Groups Most Frequently Identified by 454 Pyrosequencing

*Pseudomonas fluorescens*, a very common Gamma proteobacterium, is perhaps the best organism to illustrate common traits among the microbial community of this ecosystem. It has an extremely versatile metabolism. It is an obligate aerobe, although some strains are capable of using nitrate as a terminal electron acceptor. Critically, in terms of this study, its optimal growth temperature is 20–30 °C and so it is mesophilic. Furthermore, *Cellvibrio japonicus*, another dominant group, is a reclassification of *Pseudomonas fluorescens* subsp. *cellulosa* [67].

The majority of the most common sequences found in the library were representative of common or widespread aerobic marine species. Species in the Roseobacter clade tend to be widespread and abundant in marine environments, for example, *Roseobacter* sp. MED193. Roseobacters were first described in 1991 from marine algae. Roseobacters are well-known for processing a large amount of marine carbon and make up approximately 20% of marine bacteria [68]. *Pelagibacter ubique* is the most common microbial phylogenetic group in the open ocean, it is one of the smallest and simplest, self-replicating, and free living cells in the Alpha proteobacteria, it is heterotrophic and can reach up to ~25% of all microbial cells. During the summer, numbers can increase to ~50% of the cells in the ocean. It also has a coastal, ocean surface ecotype [3]. *Pseudomonas aeruginosa* is a common Gram-negative bacterium. It is found in all oligotropic aquatic ecosystems, which contain high-dissolved oxygen content but low nutrients. *P. aeruginosa* is therefore the most abundant organism on Earth [69]. *Congregibacter litoralis* is an aerobic anoxygenic photoheterotroph, and although these can make up over 10% of the bacterioplankonic community in oligotrophic oceans, more recent studies have shown them to be less important in open ocean and more important (over 15%) in eutrophic and mesotrophic coastal oceans [70]. *Pseudoalteromonas atlantica* is found in the ocean worldwide. It is usually found with marine eukaryotic hosts, such as crabs and seaweed. The genome of *Pseudoalteromonas atlantica* was sequenced due to its production of acidic extracellular polysaccharides during biofilm formation that show potential in element recycling, detoxification, and materials production [71]. The largely (but not entirely) psychrophilic genus *Polaribacter* is especially common in the higher latitudes of the Southern Ocean (south of the ACC basically) due to likely higher nutrients and link to eukaryotic algal primary production.

The second characteristic of the dominant groups is their success in low nutrient environments, those microorganisms with a high metabolic diversity and those identified in degradation studies using complex substrates. *Pelagibacter ubique* is characteristic of low nutrient environments [3]. The Roseobacter clade is also known for its metabolic diversity. *Rhodobacter sphaeroides* is a metabolically diverse organism suggesting a variety of methods of carbon utilization in a variety of different environments. *Maritimibacter alkaliphilus* and *Oceanicola batsensis* are known to utilize a wide range of substrates [72,73]. A number of bioremediation and bioprospecting studies have focused on degradation pathways, *Saccharophagus degradans* is a versatile marine degrader of complex polysaccharides. Gamma proteobacteria belonging to, and related to the genus *Microbulbifer* are an emerging group of complex carbohydrate-degrading marine bacteria [74]. *Marinobacter hydrocarbonoclasticus* is a halotolerant hydrocarbon degrading organism, which along with other species of *Marinobacter*, and *Ruegeria pomeroyi* plays an important role in bioremediation [73].

The optimal growth temperatures of microorganisms identified through their sequences are both mesophilic and psychrophilic [75]. *Pseudomonas putida* is found in most soil and water habitats where there is oxygen. It grows optimally at 25–30 °C. *Pseudomonas mendocina* can survive on a wide variety of substrates. Its optimal growth temperature is 30 °C [76]. *Pseudomonas fluorescens* has an optimal growth temperature of 20–30 °C and so it is mesophilic. However, *Colwellia psychrerythraea* is considered an obligate psychrophile [77].

The nitrogen cycle appears to be important. *Rosebacter denitrificans* is a purple aerobic anoxygenic phototrophic (AAP) bacterium. The highly adaptive AAPs compose more than 10% of the microbial community in some euphotic upper ocean waters and are potentially major contributors to the fixation of the greenhouse gas CO_2_ [78]. *Dinoroseobacter shibae* is an AAP bacterium capable of dissimilatory nitrate reduction. The physiological characteristics resemble those of *Roseobacter denitrificans*, but there are differences in the lipid composition. *Pseudomonas mendocina* has a denitrifying capacity which has ecological implications in the nitrogen cycle [76]. *Colwellia psychrerythraea* has coding sequences for the complete denitrification process to dinitrogen gas. This suggests *C. psychrerythrea* may play an important role in nitrogen cycling in cold, sedimentary environments [77].

The sulfur cycle less so. *Sulfitobacter* sp. NAS-14.1 is also a member of the Roseobacter clade and was cultivated from surface waters with DMSP as a sole carbon source and it comes from a group comprised of strains well known for their ability to transform inorganic and organic sulfur compounds [79]. *Roseovarius nubinhibens* is a species of aerobic dimethylsulfoniopropionate-demethylating bacterium [80].

Elsewhere, *Roseovarius* sp. 217 was isolated from a methyl halide oxidizing enrichment culture from surface seawater. This bacterium and its relatives potentially play a role in controlling fluxes of methyl halides between the ocean and the atmosphere, and the cycling of methyl halides on a global scale [81]. *Hahella chejuensis* produces abundant extracellular polysaccharides and red pigment and was originally isolated from marine sediment [82]. *Loktanella vestfoldensis* is a member of the Alpha proteobacteria isolated from the surface of the Atlantic, Antarctic lakes and biofilms [83].

### 3.14. Further Ecological Characteristics Identified by MiSeq Sequencing

Again, common and generalistic marine species predominate. Rhodobacterales bacterium HTCC2255 and HTCC2083 are members of the ubiquitous marine Roseobacter clade [84]. The genus *Octadecabacter* is also a member of the marine Roseobacter clade. The two described species of this genus, *Octadecabacter arcticus* and *Octadecabacter antarcticus*, are psychrophilic and display a bipolar distribution [85]. *Jannaschia* sp. strain CCS1 is also member of the Roseobacter lineage (Alpha Proteobacteria). CCS1 is an AAP, using bacteriochlorophyll a to harvest energy from light without the formation of oxygen. *Jannaschia* CCS1 was isolated from Pacific coastal waters on low-nutrient seawater medium. It is motile, grows slowly, and like other marine AAPs, produces bacteriochlorophyll a when grown in the dark [86]. Candidatus *Puniceispirillum marinum* IMCC1322 is the first cultured representative of the SAR116 clade in the Alpha proteobacteria. The genome contains genes for proteorhodopsin, aerobic-type carbon monoxide dehydrogenase, dimethylsulfoniopropionate demethylase, and C(1) compound metabolism. The genome information proposes the SAR116 group to be metabolic generalists in ocean nutrient cycling [87].

A key characteristic of sequences identified is the frequency of secondary metabolite genes encountered, usually indicative of the level of competition within an environment and often the target of bioprospecting studies. *Teredinibacter turnerae* optimally grows at 30–35 °C, pH 8–8.5, 0.3 M NaCl, and in elevated concentrations of Ca^2+^/Mg^2+^, which reflects its primary existence in sea water [88]. Approximately 7% of the *T. turneraes* genome are putative secondary metabolite pathways that produce antibiotics in a density similar to *Streptomyces* (the main producers of antibiotics available today) [89].

## 4. Conclusions

Recent attempts to explore marine microbial diversity and the global marine microbiome have indicated a large proportion of previously unknown diversity. In this study, although most of the more frequent sequences found were derived from common marine microorganisms, within these dominant groups, we found a proportion of genes related to secondary metabolism of potential interest in bioprospecting. Extremophiles were rare by comparison, although belonged to a number of genera which represented interesting targets from which to identify rare or novel functions. One of the most frequently encountered groups (*Colwellia psychrerythraea*) is an obligate psychrophile and the full genome is already available, but the vast majority are not obligate psychrophiles. Even the most frequently detected genes were from a relatively small proportion of the microbial population. The Pseudomonads, one of the most frequently encountered genera by both 454 pyrosequencing and MiSeq represented only 8% and 10% of sequences, respectively. The abundance of secondary metabolite genes suggested the potential for a competitive environment or indeed that key resources are used in fast growth and reproduction, however, gene expression levels are key here, not genetic potential. Nutrient cycling appears to be important, with the nitrogen cycle key, but there is evidence of the importance of sulfur cycling and methyl halide metabolism.

Previous metagenomic studies have also attempted to assess microbial abundance in similar latitudes. One of these studies [31], used metagenomics to compare bacterioplankton communities in winter with those found is summer in Antarctic coastal waters; however, their data is based only on surface waters near Palmer station (64.7742° S, 64.0531° W), south of the SACCF and not influenced by nutrient upwelling similar to that found in South Georgia, and therefore expected to harbor an altogether different type of community. Similarly, in a previous study [34], the same authors used fosmid libraries to characterize the bacterioplankton community near Palmer station during winter. In this case, inter-seasonal variation would prevent direct comparisons with our data. There are other interesting studies using metagenomics to describe the characteristics of species-specific microbial consortia, such as that published by Pucciarelli *et al.* [90]. However, none of these studies assess the specific advantages of using metagenomic approaches in bioprospecting or highlight the large proportion of genes associated with more cosmopolitan organisms. It will be interesting, nonetheless, to identify in future studies whether the proportion of mesophilic bacteria in these instances also matches that of the communities north of the SACCF.

Different methods of analysis contribute different insights to the processes under investigation. For example, neither 454 nor MiSeq, are quantitative and can only give general trends. Further, *Cellvibrio japonicus* is a soil genus that is not especially salt tolerant but as it is very close to *Pseudomonas*, it is potentially not quite correctly classified; indeed, all taxonomic descriptions based upon short amplicons, and identified via database matches need to be treated with caution. This suggests that there will be limitations with whatever approach is selected. However, the high level of agreement between independent extraction and analysis methods, gives us confidence that the results obtained are unlikely to be a result of the analysis methods alone. We deliberately adopted a dual sequencing approach to highlight the effect technique can have on the outcome of the study. Two independent extraction, sample handling and sequencing approaches gave essentially the same result. A particular strength with the production of a fosmid library is the ability to detect function through expression on selective media, without the need for knowing the target sequence of interest. Fosmids expressing a particular function of interest can be sequenced for new gene discovery. It still represents looking for a needle in a haystack, only this time using a magnet. High throughput sequencing alone will allow detection of patterns and identify those genes whose function we already know, it will also permit hypothesis generation [91], however, fosmid library construction and screening will permit expression studies. For some time there has been controversy and much discussion about bioprospecting for functional activity in Antarctic ecosystems. However, this study suggests that such activity, targeting only renewable resources, may provide novel insights and understanding of microbial function in the natural world.

## Supplementary Materials

Supplementary materials can be found at www.mdpi.com/2076-2607/4/1/8/s1.
