# Supplementary material for: Extremophiles in an Antarctic Marine Ecosystem
Source: Microorganisms. 2016 Jan 11;4(1):8. doi: 10.3390/microorganisms4010008 (PMC5029513; doi:10.3390/microorganisms4010008)
Supplement: Supplementary file 1 [file microorganisms-04-00008-s001.docx]

Supplementary Materials: Extremophiles in an Antarctic Marine Ecosystem

Iain Dickinson, William Goodall-Copestake, Michael A.S. Thorne, Thomas Schlitt, Maria Ávila-Jiménez and David A. Pearce

**Table S1**. Clones matching the sequences detected within the 20 quality control fosmids.

| **Metagenome** | **Clone** |
| --- | --- |
| Global-Ocean Sampling | GS-32-01-01-1P3-1P6KB |
|  | GS-35-01-01-1P5KB |
|  | GS-30-02-01-1P0-1P3KB |
|  | GS-27-01-01-1P8-2P0KB |
|  | GS-28-01-01-1P6-2P0KB |
|  | GS-33-01-01-1P3-1P8KB |
| Marine metagenome genomic clones | 1061005919604 5’ |
|  | 1061002384549 5’ |
|  | 1061001790330 5’ |
|  | 1061005446189 3’ |
|  | 1061002401470 5’ |
|  | 1061006194546 5’ |
| Ionian sea marine metagenome | akm3am44r Km3-3010m |
|  | akm3bw24r Km3-3010m |
|  | akm3am44r Km3-3010m |

**Table S2.** Number of bacterial genera by 454 pyrosequencing (with percentage abundance in parenthesis).

| **Genera** | **Abundance** |
| --- | --- |
| Proteobacteria | 165 (88.82%) |
| Alpha | 50 (41.36%) |
| Beta | 24 (5.6%) |
| Delta | 18 (1.44%) |
| Epsilon | 8 (0.3%) |
| Gamma | 64 (39.94%) |
| Unclassified | (0.18%). |
| Bacteroidetes | 18 (5.6%) |
| Actinobacteria | 46 (1.94%) |
| Firmicutes | 33 (1.27%) |
| Cyanobacteria | 12 (0.79%) |
| Acidobacteria | 2 (0.27%) |
| Chlorobi | 5 (0.27%) |
| Planctomycetes | 3 (0.23%) |
| Chloroflexi | 6 (0.21%) |
| Deinococcus-Thermus | 3 (0.18%) |
| Spirochaetes | 3 (0.15%) |
| Thermotogae | 4 (0.10%) |
| Verrucomicrobia | 3 (0.10%) |
| Fusobacteria | 4 (0.07%) |
| Deferribacteres | 1 (0.04%) |
| Aquificae | 3 (0.03%) |
| Chlamydiae | 3 (0.03%) |
| Synergistetes | 2 (0.02%) |
| Tenericutes | 6 (0.02%) |
| Dictyoglomi | 1 (0.01%) |
| Elusimicrobia | 1 (0.01%) |
| Unclassified | (0.01%) |

**Table S3.** The top ten archaeal species by abundance using 454 Pyrosequencing.

| **Species** | **Abundance** |
| --- | --- |
| *Methanosarcina acetivorans* | 165 |
| *Haloarcula marismortui* | 155 |
| *Methanococcus maripaludis* | 154 |
| *Natronomonas pharaonis* | 130 |
| *Methanothermobacter thermautotrophicus* | 111 |
| *Halogeometricum borinquense* | 98 |
| *Methanobrevibacter smithii* | 73 |
| *Methanosarcina barkeri* | 73 |
| *Pyrococcus horikoshii* | 72 |
| *Methanosarcina mazei* | 61 |

**Table S4.** Gene ontology by 454 pyrosequencing and MiSeq.

| **Function of Gene** | **Abundance in 454 Data** | **Abundance in MISEQ Data** |
| --- | --- | --- |
| Amino Acids and Derivatives | 125634 | 735503 |
| Carbohydrates | 137917 | 1497759 |
| Cell Divison and Cell Cycle | 12888 | 80155 |
| Cell Wall and Capsule | 42468 | 503023 |
| Clustering-based subsytems | 157986 | 1119354 |
| Cofactors, Vitamins, Prosthetic Groups, Pigments | 71347 | 433115 |
| DNA Metabolism | 36818 | 271635 |
| Dormancy and Sporulation | 1878 | 11165 |
| Fatty Acids, Lipids, and Isoprenoids | 52748 | 241736 |
| Iron acquisition and metabolism | 13238 | 130881 |
| Membrane Transport | 27085 | 267344 |
| Metabolism of Aromatic Compounds | 20571 | 92333 |
| Miscellaneous | 100789 | 548018 |
| Motility and Chemotaxis | 16051 | 106429 |
| Nitrogen Metabolism | 7399 | 113417 |
| Nucleosides and Nucleotides | 26810 | 200924 |
| Phages, Prophages, Transposable elements, Plasmids | 10911 | 128540 |
| Phosphorus Metabolism | 4623 | 75667 |
| Photosynthesis | 2307 | 2109 |
| Potassium metabolism | 2603 | 78317 |
| Protein Metabolism | 77132 | 409040 |
| Regulation and Cell signaling | 11528 | 263081 |
| Respiration | 28736 | 429410 |
| RNA Metabolism | 69752 | 225760 |
| Secondary Metabolism | 3181 | 47330 |
| Stress Response | 30024 | 300517 |
| Sulfur Metabolism | 8547 | 81598 |
| Virulence, Disease and Defense | 14757 | 258210 |

**Table S5.** The number of bacterial genera by MISEQ (with percentage abundance in parenthesis).

| **Genera** | **Abundance** |
| --- | --- |
| Proteobacteria | 299 (84.75%) |
| Alpha | 93 (29.33%) |
| Beta | 53 (3.14%) |
| Delta | 28 (0.56%) |
| Epsilon | 13 (0.13%) |
| Gamma | 111 (51.4%) |
| Zeta | 1 (0.01%) |
| Unclassified | (0.17%). |
| Bacteroidetes | 41 (4.27%) |
| Actinobacteria | 71 (0.99%) |
| Cyanobacteria | 24 (0.65%) |
| Acidobacteria | 5 (0.21%) |
| Gemmatimonadetes | 1 (0.13%) |
| Nitrospirae | 4 (0.13%) |
| Verrucomicrobia | 8 (0.09%) |
| Chlorobi | 5 (0.08%) |
| Chloroflexi | 11 (0.08%) |
| Planctomycetes | 8 (0.08%) |
| Deinococcus-Thermus | 5 (0.07%) |
| Tenericutes | 5 (0.07%) |
| Spirochaetes | 5 (0.06%) |
| Fusobacteria | 4 (0.03%) |
| Chlamydiae | 5 (0.02%) |
| Lentisphaerae | 2 (0.02%) |
| Thermotogae | 7 (0.02%) |
| Chrysiogenetes | 1 (0.01%) |
| Deferribacteres | 3 (0.01%) |
| Synergistetes | 8 (0.01%) |
| Elusimicrobia | 2 (0.001%) |
| Fibrobacteres | 1 (0.001%) |
| Poribacteria | 1 (0.001%) |
| Unclassified | (1.5%) |

**Table S6.** The top ten archaeal species by abundance using MiSeq.

| **Species** | **Abundance** |
| --- | --- |
| *Methanococcus maripaludis* | 54 |
| *Methanothermobacter thermautotrophicus* | 50 |
| *Methanosarcina mazei* | 46 |
| uncultured marine crenarchaeote HF4000_APKG2o16 | 41 |
| *Methanosarcina acetivorans* | 38 |
| *Haloquadratum walsbyi* | 35 |
| *Ignicoccus hospitalis* | 34 |
| *Natronomonas pharaonis* | 34 |
| *Haloferax volcanii* | 26 |
| *Methanococcoides burtonii* | 26 |

**Table S7.** The Cyanobacteria identified in this study.

| **Identified Cyanobacteri** |
| --- |
| *Acaryochloris marina* |
| *Anabaena variabilis* |
| *Arthrospira maxima* |
| *Arthrospira platensis* |
| *Arthrospira* sp. |
| *Crocosphaera watsonii* |
| *Cyanobium* sp. (8 species) |
| *Cylindrospermopsis raciborskii* |
| *Dactylococcopsis salina* |
| *Gloeobacter violaceus* |
| *Lyngbya* sp. |
| *Microcoleus chthonoplastes* |
| *Microcystis aeruginosa* |
| *Nodularia spumigena* |
| *Nostoc* sp. (2 species) |
| *Oscillatoria* sp. |
| *Prochlorococcus marinus* |
| *Raphidiopsis brookii* |
| *Synechococcus* sp. (17 species) |
| *Synechocystis* sp. |
| *Thermosynechococcus elongates* |
| *Tolypothrix* sp. |
| *Trichodesmium erythraeum* |
| *Trichormus azollae* |
